# Miscellany

**Published:** 1904-09

**Authors:** 


### Miscellany.

Poisoning by Corrosives.—Important as is the employment of a chemical antidote, there is something more important in view of the power of the concentrated poison and the rapidity of its action. For, in order to apply the chemical antidote, it must be ascertained just what poison has been taken. This will consume time and delay treatment during the period of greatest danger to the tissues. The important thing to do at once is to limit the corrosive action of the poison. The author believes that the most important thing, which can always be done most readily, is to dilute the poison largely by a copious draft of water, and this without reference to the character of the poison. By this means we stop further corrosion by converting the strong acid, alkali, or salt into a comparatively harmless dilution of it. The selection and use of the chemical antidote can then follow as a matter secondary in point of time.

The poison having been diluted, it should then be neutralized as quickly as possible by the chemical antidote. Corresponding closely to the three groups of poison under consideration, the chief antidotes of practical value comprise the following three groups: (1) Dilute alkalies; (2) dilute acids; (3) albuminous substances.

For the group of caustic alkalies the proper antidote would be a dilute acid, preferably vegetable acids such as vinegar or lemon-juice, but any acid largely diluted may be employed. In case the poison is stronger water of ammonia, a volatile acid is needed in addition to neutralize the irritating vapor in the air-passages. Strong acetic acid by inhalation will answer this purpose.

The coagulant group will all be neutralized by egg albumin, or, as substitutes for it, by milk or flour paste. In addition a mild alkali is indicated in order to neutralize the liberated acid, except in case of carbolic acid poisoning, which requires special treatment, in addition to the use of albumin. The special antidotes for agents of this group in addition to albumin will be here noted: For carbolic acid alcohol has come to be employed, its effect being probably more upon the tissues than upon the poison itself. Soluble sulphates are also employed and continued for some time in order to avert within the system the formation of the products which are irritating to the kidneys. For silver nitrate, sodium chloride is an excellent additional antidote.

A marked peculiarity of carbolic acid poisoning is the rapidity of systemic effect, seemingly out of proportion to the injury done to the tissues. Death from carbolic acid will very frequently ensue within an hour's time, showing that either severe shock to the nervous system or very rapid systemic poisoning becomes a factor.

After the proper chemical antidotes have been administered emesis should be favored, unless already excessive, by copious drafts of warm water. When the poison or its compounds have been removed, demulcents should be employed freely to soothe the corroded and irritated tissues, and they should be allowed to remain within the stomach in sufficient bulk to prevent further damage to its walls by friction. Either of the ordinary classes of demulcents may be employed, which include the oils, mucilages, and albuminous substances. These measures will constitute the immediate treatment of poisoning by corrosives and the attendant damage to the tissues. In addition it may be necessary to employ morphine hypodermically in order to relieve pain and lessen peristalsis, and to employ stimulants judiciously. Perfect rest in bed for a number of days is enjoined, with attention to details as each case may require.

Although arsenic does not belong to the class of corrosives, its destructive action permits reference here to its antidotal treatment. Its chemical antidote is easily prepared, and the process should be indelibly fixed in every practitioner's mind, as it must be fresh when needed. The usual antidote is the hydrated oxide of iron, obtained by mixing any solution of a ferric salt with water of ammonia, both having been previously diluted. Either tincture of the chloride or Monsel's solution may be employed for the purpose, and the water of ammonia may be substituted by hydrated magnesia, in which case the product is the equally valuable hydrated oxide of iron with magnesia. The antidote, suspended in water, may be given freely.—*Therapeutic Gazette.*

---

A Modified Needle for Pressure Anæsthesia.—I have modified the form of the ordinary long, re-enforced hypodermic needle, so that with it the local anæsthetic used by me in extracting is made to do all and more than I could do with the saturated solution following the common method. In this manner I use analgine, which contains but one-half of one per cent. of cocaine. I dare say any similar solution would give the same result. I find this method especially adapted to the treatment of children's teeth, for, as a rule, the dam cannot be used, and all danger from the solution escaping into the mouth is eliminated. Every dentist will find in his cabinet abundant material with which to make the points to which I have referred and now will describe.

From the base of an old point make a section which will be bell-shaped. Selecting another needle, remove the steel point and thread the end of the re-enforcement to screw into the bell-shaped point just described, and the instrument is complete. Three of these points have answered in the majority of cases. The angles may be changed at will by slight bending of the stems. It is unnecessary to say that these points are used with the regular syringe. In using this instrument apply the dam where possible, as an aseptic precaution. Dry the cavity as well as permissible and apply sanguestine chloride [lily] or adrenalin, wiping the cavity with carbolic acid. Now from a sheet of unvulcanized rubber cut a disk large enough to amply cover the point of exposure. With rubber-dam punch make a small hole in the centre of this disk of rubber, which you will now slightly coat with cavitine varnish or chloropercha and place over exposure so that the central opening is directly over the point of exposure. It only remains now to make application of the bell-shaped point over the hole in the disk and gradually get the pressure necessary to force the solution through the entire pulp. It is now almost a year since I adopted this method, and trust that others of the profession may have as good results as I have had in its application.—Dr. J. A. Johnson.

---

Temporary Filling.—Absorbent cotton saturated with cement which has been mixed to a creamy consistency makes an excellent temporary filling-material. It will last for months. If only required for a short time, nearly fill the cavity with dry cotton before inserting the plug. This will facilitate the removal of the filling when it becomes necessary.—R. E. Sparks, Kingston, Ont.

